# Relationship between Bone-Specific Physical Activity Scores and Measures for Body Composition and Bone Mineral Density in Healthy Young College Women

**DOI:** 10.1371/journal.pone.0162127

**Published:** 2016-09-02

**Authors:** SoJung Kim, Wi-Young So, Jooyoung Kim, Dong Jun Sung

**Affiliations:** 1 Department of Physical Therapy, College of Health Sciences, University of Massachusetts, Lowell, Massachusetts, 01854, United States of America; 2 Sports and Health Care Major, College of Humanities and Arts, Korea National University of Transportation, Chungju-si, 27469, Korea; 3 Health and Rehabilitation Major, Kookmin University, Seoul, 02707, Korea; 4 Division of Sport Science, College of Science and Technology, Konkuk University, Chungju-si, 27478, Korea; Leeds Beckett University, UNITED KINGDOM

## Abstract

**Objective:**

The purpose of this cross-sectional study was to investigate the relationship between bone-specific physical activity (BPAQ) scores, body composition, and bone mineral density (BMD) in healthy young college women.

**Methods:**

Seventy-three college women (21.7 ± 1.8 years; 162.1 ± 4.6 cm; 53.9 ± 5.8 kg) between the ages of 19 and 26 years were recruited from the universities in Seoul and Gyeonggi province, South Korea. We used dual energy X-ray absorptiometry to measure the lumbar spine (L2-L4) and proximal femur BMD (left side; total hip, femoral neck). The BPAQ scores (past, pBPAQ; current, cBPAQ; total, tBPAQ) were used to obtain a comprehensive account of lifetime physical activity related to bone health. We used X-scan plus II instrumentation to measure height (cm), weight (kg), fat free mass (FFM, kg), percent body fat (%), and body mass index (BMI). Participants were asked to record their 24-hour food intake in a questionnaire.

**Results:**

There were positive correlations between BPAQ scores and total hip (pBPAQ r = 0.308, p = 0.008; tBPAQ, r = 0.286, p = 0.014) and FN BMD (pBPAQ r = 0.309, p = 0.008; tBPAQ, r = 0.311, p = 0.007), while no significant relationships were found in cBPAQ (p > 0.05). When FFM, Vitamin D intake, cBPAQ, pBPAQ, and tBPAQ were included in a stepwise multiple linear regression analysis, FFM and pBPAQ were predictors of total hip, accounting for 16% (p = 0.024), while FFM and tBPAQ predicted 14% of the variance in FN (p = 0.015). Only FFM predicted 15% of the variance in L2-L4 (p = 0.004). There was a positive correlation between Vitamin D intake and L2-L4 (p = 0.025), but other dietary intakes variables were not significant (p > 0.05).

**Conclusions:**

BPAQ-derived physical activity scores and FFM were positively associated with total hip and FN BMD in healthy young college women. Our study suggests that osteoporosis awareness and effective bone healthy behaviors for college women are required to prevent serious bone diseases later in life.

## Introduction

Osteoporosis is the most common type of bone disease characterized by low bone mineral density (BMD), high bone turnover, and microarchitecture deterioration [1]. It is well known that peak bone mass is reached by the end of the third decade [2] and increasing BMD levels is crucial for preventing osteoporosis during the early adulthood. High-impact exercises, including jumping, dynamic movements, and resistance training are recommended to increase BMD in premenopausal women [3, 4]. In addition, maintaining optimal muscle mass is an important factor determining bone health in young adults [5, 6]. Other factors influencing BMD in college women have been unhealthy weight control behaviors [7, 8], low body mass index [9, 10] and low Vitamin D intake [10], low calcium intake [11], and lifestyle effects [11–13]. Due to the lack of awareness or education for serious bone diseases such as osteoporosis among female college students [14, 15], the last opportunity to build strong bones for life may not be importantly recognized by this population. As it turns out, mechanical loading of the skeleton during high impact weight-bearing activities (e.g., running, jumping) has been shown to improve bone mass in humans [16, 17].

A variety of physical activity assessment tools (e.g., questionnaires, pedometers, and accelerometers) have been used to predict bone strength in diverse population groups [18–22]. Among these methods, the bone-specific physical activity questionnaire (BPAQ) is a relatively simple method to account for the relationship between physical activity associated with bone loading and BMD as measured by dual energy X-ray absorptiometry (DXA) and calcaneal broadband ultrasound attenuation (BUA) [18]. The BPAQ algorithms are becoming more widely used in children [20, 23, 24], adolescents [25, 26], young adults [18, 24], and clinical populations [27, 28] to determine their relation to areal BMD (aBMD), geometry, and bone architecture. The results of previous studies have shown BPAQ able to predict femoral neck (FN), lumbar spine, and whole body aBMD (g/cm^2^) in young men (24.5±2.9 years) [18] and in healthy middle-aged and older men (60.6±6.0 years) [28], while the past BPAQ score predicted an index of bone strength at the heel for healthy young women (25.1±2.8years) [18]. However, a study by Farr et al. [20] found that BPAQ score was not significantly associated with bone strength index (BSI) and strength strain index (SSI) at femur and tibia sites as measured by peripheral quantitative computed tomography (pQCT) in girls (10.7±1.1 years) compared with past physical activity questionnaire (PYPAQ). The BPAQ algorithms has been validated in a small number of young adults (20 men and 20 women) [18] and BPAQ’s relation to BMD in healthy college women has not been reported.

The purpose of this cross-sectional study was to investigate the relationship between BPAQ-derived physical activity, body composition, and BMD in Korean college women. We hypothesized that the BPAQ scores would have positive associations with total hip, FN, and L2-L4 aBMD in healthy young college women.

## Materials and Methods

### Participants

Seventy-three college women between the ages of 19 and 26 years were recruited from the universities in Seoul and Gyeonggi province, South Korea. Healthy individuals who had regular menstrual cycles participated in the study. The participants were excluded if they were smokers, pregnant, on hormonal birth control, taking antihypertensive drugs or any medication that could affect BMD. All participants visited the Seoul Sok Medical Center to complete an informed consent form, the health and menstrual history questionnaires along with bone scans and body composition assessments.

This study was approved by Korean Ministry of Health and Welfare Research Ethics Committee and written informed consent was obtained from each participant.

### Body composition

We used X-scan plus II (Hospital body Composition Analyzer, Jawon Medical, Gyeongsan, Korea) to measure height (cm), weight (kg), fat free mass (FFM) (kg), % body fat (%), and body mass index (BMI). To avoid measurement errors, the participants were asked not to drink alcohol for 48 hours before the testing or participate in any vigorous exercise 12 hours prior to the testing. They were also asked not to have any meals including beverages 4 hours before the testing. All participants changed into the scrubs that were provided by the Seoul Sok Medical Center. The participants stood on the measure scale barefoot and held the measurement bars with both hands and open arms.

### Bone Mineral Density

We used dual energy X-ray absorptiometry (DXA, GE Lunar Prodigy) to measure L2-L4 and the left side of proximal femur BMD (total hip, femoral neck; FN). The DXA was calibrated daily following the Quality Assurance and qualified technicians conducted all procedures during the study period. The participants removed all metal, plastic objects or other high density objects associated with the participant’s clothes. The participants were asked to lie down on the DXA table in the supine position. The standard scan mode was determined by the level of participant’s umbilicus (Thick, >25 cm; Standard, 13–25 cm; and Thin, <13 cm). For lumbar spine measurement, an appropriate positioning block was chosen and placed under the participant’s legs. For the proximal femur of non-dominant side leg, the participant’ feet were secured by the DualFemur^TM^ positioner to maintain the appropriate internal rotation of trochanter or 1 cm inferior to the pubic symphysis in the midline of the thigh. Two qualified technicians performed all scans and analyses using encore 2002 Software (GE Lunar Prodigy).

### 24 hours recall

Participants were asked to complete a questionnaire, recalling food intake for the past 24 hours, and this included listing food/drink items with brand names, the amount ingested, and method of preparation with as much specificity as possible. We also obtained information regarding the supplements taken by the participants. A qualified dietitian analyzed total caloric intake (kcal), protein (g), carbohydrate (g), fat (g), Vitamin D (mcg), calcium (mg), and magnesium (mg) using the Computer Aided Nutritional analysis program (CAN-Pro 4.0, The Korean Nutrition Society).

### Bone-specific Physical Activity Questionnaire

The BPAQ assessment instrument was designed to estimate levels of physical activities that impose mechanical loads on the skeleton [18]. The BPAQ consisted of three independent sections: the past period (pBPAQ, from one year of age to the current age), the current period (cBPAQ, previous 12 months), and the total period (tBPAQ, average of pBPAQ and cBPAQ) [28]. Participants were asked to complete the BPAQ questionnaire and a qualified researcher analyzed all values using an online BPAQ calculator (www.fithdysign.com/BPAQ/). Algorithms used to analyze BPAQ responses have been described in detail [18].

### Statistical analyses

We performed all analyses using SPSS for Windows version 22 (SPSS Inc., Chicago, IL, USA) and data are reported at mean ± SD. Pearson’s correlation tests were used to examine the association between the BPAQ scores (pBPAQ, cBPAQ, and tBPAQ), body composition, dietary intake and BMD. We performed stepwise multiple regression analyses to determine the variables that predict variance in BMD. The predictor variables included FFM, Vitamin D intake, BPAQ scores, and BMD (total hip, FN, L2-L4). We set the level of significance at p < 0.05.

## Results

We provide physical characteristics, body composition variables, BPAQ scores, dietary intake estimates and BMD for study participants in Table 1.

**Table 1:** Descriptive Data for the Study Population (N = 73).

Variables	Mean ± SD	Range
**Age (years)**	21.7 ± 1.8	19.0–25.8
**Height (cm)**	162.1 ± 4.6	151.7–172.4
**Weight (kg)**	53.9 ± 5.8	41.8–68.6
**Menarche age (years)**	12.4 ± 1.4	9.0–16.0
**BMI (kg/m**^**2**^**)**	21 ± 2	16–26
**FFM (kg)**	40.0 ± 2.9	33.9–47.4
**% body fat (%)**	25.4 ± 4.11	15.2–35.6
**BPAQ**		
**Current**	3.8 ± 7.1	0.3–42.7
**Past**	40.5 ± 10.8	11.2–83.6
**Total**	22.2 ± 6.7	6.0–49.3
**Dietary Intake**		
**Total caloric intake (kcal)**	1578.3 ± 478.1	736.7–2885.9
**Protein (g)**	63.0 ± 29.8	16.5–192.1
**Carbohydrate (g)**	211.3 ± 66.1	78.4–380.3
**Fat (g)**	54.5 ± 23.8	15.0–130.5
**Vitamin D (mcg)**	3.7 ± 3.9	0.0–18.4
**Calcium (mg)**	432.3 ± 236.7	72.3–1070.3
**Magnesium (mg)**	77.6 ± 54.0	0.0–238.7
**BMD (g/cm**^**2**^**)**		
**Total hip**	0.979 ± 0.109	0.727–1.257
**FN**	0.969 ± 0.113	0.731–1.287
**L2-L4**	1.167 ± 0.118	0.924–1.262

BMI: body mass index, FFM: fat free mass, BPAQ: bone-specific physical activity score, BMD: bone mineral density, FN: femoral neck

We found that none of the participants had less than or equal to a -2.5 T-score (classified as osteoporosis) for total hip, FN, and L2-L4 (Table 2).

**Table 2:** Classification of the Participants by Bone Status (N = 73).

T-score	Total hip	FN	L2-L4
	n(%)	n(%)	n(%)
**Normal (T-score ≥ -1.0)**	63(86)	55(75.3)	53(72.6)
**Osteopenia (–2.5 < T-score < -1.0)**	10(14)	18(24.7)	20(27.4)
**Osteoporosis (T-score ≤ -2.5)**	0(0)	0(0)	0(0)

FN: femoral neck

**Fig 1:**
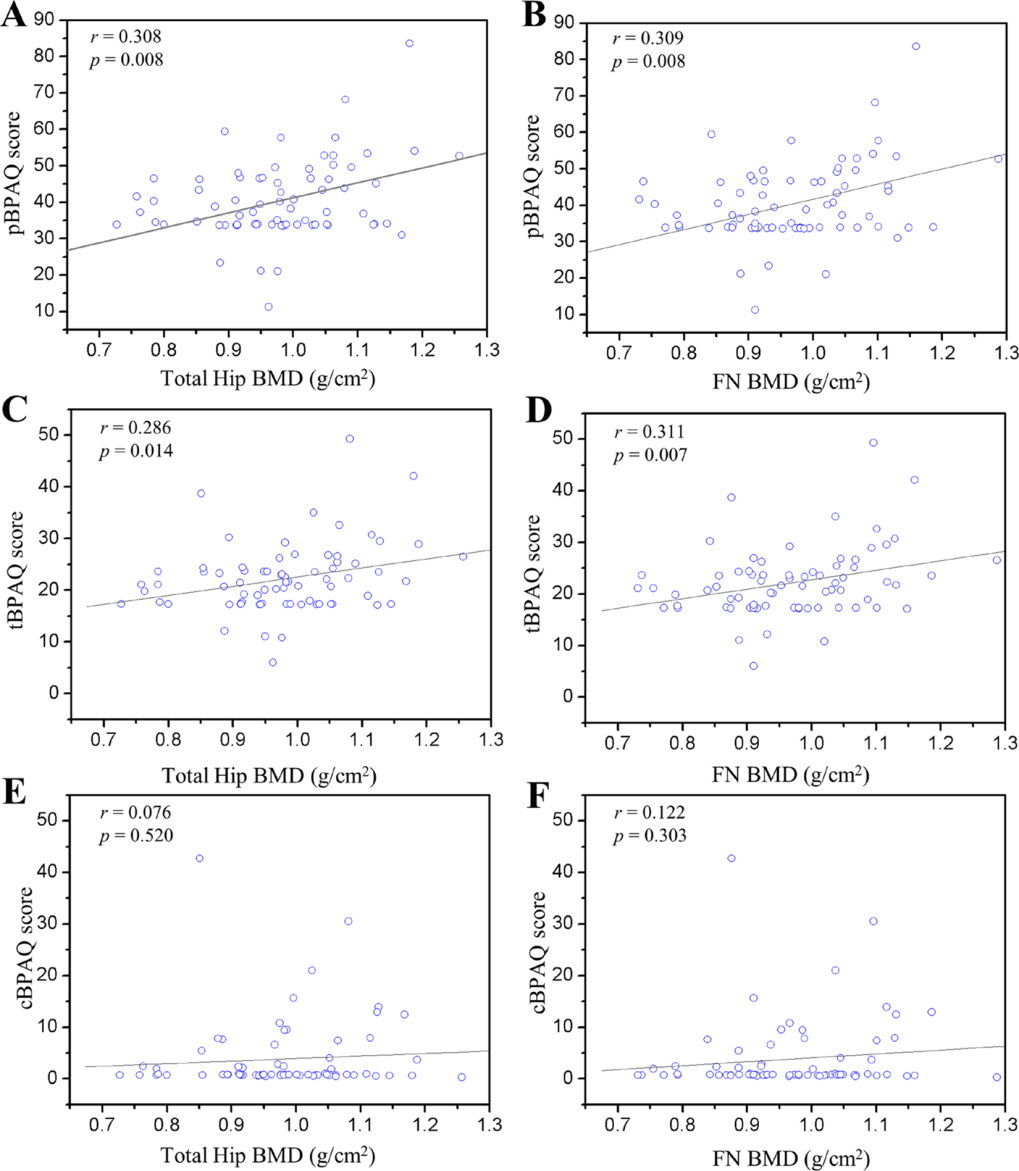
Correlation of BPAQ scores with total hip and FN BMD. **A,** pBPAQ score with total hip BMD (r = 0.308, p = 0.008). **B**, FN BMD (r = 0.309, p = 0.008)**. C**, cBPAQ score with total hip BMD (r = 0.286, p = 0.014). **D**, FN BMD (r = 0.311, p = 0.007). **E**, cBPAQ score with total hip BMD (r = 0.076, p = 0.520). **F**, cBPAQ score with FN BMD (r = 0.122, p = 0.303). FN: femoral neck

There were positive correlations between BPAQ scores and total hip (pBPAQ r = 0.308, p = 0.008; tBPAQ, r = 0.286, p = 0.014) and FN BMD (pBPAQ r = 0.309, p = 0.008; tBPAQ, r = 0.311, p = 0.007) while no significant relationship was found in cBPAQ (total hip, r = 0.076, p = 0.520; FN, r = 0.122, p = 0.303) (Fig 1). No significant relationships were found between cBPAQ (r = 0.045, p = 0.707), pBPAQ (r = 0.174, p = 0.141), tBPAQ (r = 0.116, p = 0.330) and L2-L4. When FFM, Vitamin D intake, cBPAQ, pBPAQ, and tBPAQ were included in a stepwise multiple linear regression analysis, FFM and pBPAQ were predictors of total hip, accounting for 16% (p = 0.024), while FFM and tBPAQ predicted 14% of the variance in FN (p = 0.015). Only FFM predicted 15% of the variance in L2-L4 (p = 0.004).

The relationships between body composition variables and total hip, FN, and L2-L4 BMD are shown in Table 3.

**Table 3:** Correlation of Body Composition Variables with BMD (N = 73).

Variables	Total hip	FN	L2-L4
**Weight (kg)**	.297*	.273*	.343**
**BMI (kg/m**^**2**^**)**	.289*	.270*	.283*
**FFM (kg)**	.278*	.238*	.396***
**% body fat (%)**	.215	.221	.158

BMI: body mass index, FFM: fat free mass

*p < 0.05

**p < 0.01

***p = 0.001

Significant positive relationships were found between weight, BMI, FFM, and total hip BMD (p < 0.05). Pearson’s correlation tests showed positive relationships between weight, BMI, FFM, and BMD at the left side of FN (p < 0.05). Also, weight (p < 0.01), BMI (p < 0.05), and FFM (p = 0.001) were positively related to the L2-L4. There were no correlations between % body fat and total hip, FN, and L2-L4 (p > 0.05). There was a positive correlation between Vitamin D and L2-L4 (p = 0.025). All other dietary intake variables did not have significant relationships with BMD variables (p > 0.05) (Table 4).

**Table 4:** Correlation of Dietary Intake Variables with BMD (N = 73).

Variables	Total hip	FN	L2-L4
**Total caloric intake (kcal)**	.142	.052	.115
**Protein (g)**	.165	.125	.208
**Carbohydrate (g)**	.060	.025	.030
**Fat (g)**	.165	.036	.105
**Magnesium (mg)**	-.027	-.076	.084
**Vitamin D (mcg)**	.065	.076	.262*
**Calcium (mg)**	.062	.041	-.107

*p < 0.05

## Discussion

The aim of this cross-sectional study was to determine the relationship between BPAQ scores and body composition, and BMD in healthy young college women. Our study found that BPAQ scores were positively related to total hip and FN BMD and no correlations were detected in L2-L4. Also, the past and total component of BPAQ scores and FFM were significant predictors of total hip (16%) and FN BMD (14%).

Weeks and Beck [18] reported that cBPAQ was a significant predictor of variance in FN, lumbar spine, and whole body aBMD in young men (n = 20), but not in women (n = 20). However, our study showed that cBPAQ score was not sensitive to predict any relevant bone sites. Bolam et al. [28] found that FN aBMD was positively related to all BPAQ scores, but total hip and whole body aBMD were related to pBPAQ and tBPAQ in 36 healthy older men (n = 36). Farr et al. [20] reported that BPAQ scores were not significantly associated with BSI or SSI at femur and tibia sites, measured by pQCT in girls. A study by Kindler et al. [25] found that BPAQ scores were correlated with all mid-tibia cortical bone architecture at the non-dominant leg (r = 0.41–0.51), assessed by magnetic resonance imaging (MRI) and most aBMD (r = 0.47–0.53) measures in non-Hispanic white adolescent females (n = 24).

The inconsistent results may be due to differences in bone measurements (DXA, pQCT, and MRI) and bone parameters (aBMD, geometry, and microstructure). In addition, most previous BPAQ studies for women have used relatively small sample sizes and correlations between the BPAQ scores and aBMD at common osteoporotic fracture sites may be age-dependent (children, adolescent, and young women) [28]. In the current study, all three BPAQ scores did not predict L2-L4 aBMD, and similar results were found in older men [28] and young women [18]. As a result, BPAQ scores (past and total) would be strong predictors of FN and total hip aBMD in healthy young college women.

Based on the World Health Organization (WHO) criteria for diagnosis of osteoporosis [29], in the current study, 24.7% and 27.4% of the participants were classified as osteopenia FN and L2-L4, respectively. Compared with previous results [30, 31], our participants had a lower risk of osteopenia bones (24.7%-27.4% *vs*. 33.5%-45.9%), but over 70% of the participants with normal T-score (T-score ≥ -1.0) had relatively lower mean T-scores (-0.37). Also, our participants showed lower calcium (432.3 ± 236.7 mg) and Vitamin D intake (3.7 ± 3.9 mcg) and these results did not meet the recommended dietary allowances for calcium (1000 mg/day) and Vitamin D intake(15 mcg/day) [32]. Previous studies have shown similar results, suggesting that college women with inadequate calcium consumption and Vitamin D intake increased the risk of fractures and osteoporosis [11, 30, 31]. Considering that up to 90% of peak bone mass is obtained by age 18 in girls, the importance of osteoporosis awareness and effective bone healthy behaviors for college women would be needed to prevent serious bone diseases in later life.

In the current study, weight, BMI, and FFM were positively related to the total hip, FN, and L2-L4, but % body fat was not significantly correlated. These findings are consistent with previous studies showing lower BMI was associated with lower BMD [33, 34]. Also, among body composition variables, our study showed that the FFM was a relevant factor related to bone sites and was strongly associated with L2-L4 (r = 0.396, p = 0.001). We found that none of the bone measurement sites was correlated with % body fat. Lee et al. [35] reported that lean body mass, muscle mass, and fat mass had positive relationships with lumbar spine and FN aBMD, but % body fat was only related to FN and not lumbar spine in young healthy women. However, another study showed that fat mass and BMD had a negative correlation in premenopausal women [36]. Similarly, fat mass showed a negative association with FN and femur aBMD in healthy Caucasian women [37]. Park et al. [38] suggested that both lean mass and fat mass had positive genetic correlations, but increased lean mass would be more beneficial for BMD in middle-aged Korean women. The relationships between fat components and BMD have shown conflicting results, suggesting that the age, gender, and menopausal status would differently affect the results [36]. Further studies are needed to determine effects of fat on bone density and provide healthy body composition of maintaining bone health in young college women.

There are several limitations to our study. Although a qualified researcher in detail explained and assisted with the BPAQ questionnaire, the participants’ recall errors would affect the individual BPAQ scores. We also used a 24-hour food intake recall questionnaire to identify dietary factors that affect BMD in healthy young college women, but this method would not represent participants’ normal diets. For future study, 3-day dietary log (two days during the week and one day during the weekend) would be more useful to represent normal dietary intake in this population. While most previous studies have widely used DXA to measure body composition components to investigate their relation to BMD, X-scan plus II was used in the current study. Even though the BIA has provided a relatively accurate prediction of % body fat in individuals with normal weight, overweight or obesity [39] and FFM in healthy Asian individuals [40], several studies have reported that it is influenced by different ethnic groups [41] and gender [42]. Therefore, ethnicity, age, and gender might be considerable factors to compare results from different body composition measurement tools.

## Conclusion

Our findings indicated that BPAQ scores were positively related to total hip and FN BMD and no correlations were detected in L2-L4. Also, BPAQ scores (past and total) and FFM were significant predictors of the proximal femur of non-dominant side leg. Except for % body fat, body composition components were positively related to the relevant bone sites. Our study suggests that osteoporosis awareness and effective bone healthy behaviors for college women are required to prevent serious bone diseases later in life.

## Supporting Information

S1 File Raw data of Fig 1. (XLSX)
